# Does unemployment lead to greater levels of loneliness? A systematic review

**DOI:** 10.1016/j.socscimed.2021.114339

**Published:** 2021-10

**Authors:** N. Morrish, A. Medina-Lara

**Affiliations:** Health Economics Group, Institute of Health Research, University of Exeter Medical School, University of Exeter, Exeter, UK

**Keywords:** Loneliness, Unemployment, Employment, Working-age, Adults, Systematic review, Bi-directional

## Abstract

There is evidence that loneliness and unemployment each have a negative impact on public health. Both are experienced across the life course and are of increasing concern in light of the COVID-19 pandemic. This review seeks to examine the strength and direction of the relationship between loneliness and unemployment in working age individuals, and in particular the potential for a self-reinforcing cycle with combined healthcare outcomes. A systematic search was undertaken in Medline, PubMed, PsycINFO, Embase and EconLit from inception to December 2020. PRISMA reporting guidelines were followed throughout this review, study quality was assessed using the Joanna Briggs Institute checklist and results were summarised in a narrative synthesis. English language studies evaluating the relationship between loneliness and unemployment in higher income western countries were included. Thirty-seven studies were identified; 30 cross-sectional and 7 longitudinal. Loneliness was measured by a direct question or loneliness scale while unemployment was self-reported or retrieved from a national register. A positive association between unemployment and increased loneliness was observed across all studies. Thus, across the life-course a clear yet complex relationship exists between unemployment and greater experience of loneliness. The magnitude of this relationship increases with the severity of loneliness and appears to peak at age 30–34 and 50–59. Logistic regression provided the greatest consistency at statistical significance revealing at least a 40% increase in the likelihood of reporting loneliness when unemployed. Recent longitudinal studies identified in this review found higher levels of loneliness following job loss, but also that loneliness was predictive of unemployment suggesting potential bi-directionality in the relationship. This bi-directionality may create a multiplier effect between loneliness and unemployment to form a self-reinforcing relationship and greater health concerns for those most at risk. Thus, review findings suggest the need for cross-sector awareness and intervention to tackle both loneliness and unemployment.

## Highlights

• Unemployment is related to feeling lonely.
• Stronger association of unemployment with severe experience of loneliness.
• Greatest connection between loneliness and unemployment at age 30–34 and 50-59.
• Potential for a bi-directional relationship between loneliness and unemployment.
• Greater understanding is needed in the face of COVID-19 recovery.

## 1 Introduction

Loneliness has been described as a ‘public health epidemic’ (Royal College of General Practitioners, 2018) and is of increasing concern in light of the current COVID-19 pandemic. Loneliness captures the deficit between desired and actual social relationships (Fulton and Jupp, 2015) and is increasingly acknowledged to be associated with health outcomes and unemployment (Leigh-Hunt et al., 2017; Matthews et al., 2018). While the literature on health outcomes related to loneliness has grown in recent years, particularly concerning older adults, understanding of the relationship between loneliness and wider socio-economic or economic factors, such as unemployment, remains limited (Leigh-Hunt et al., 2017). This remains the case despite recognition of greater loneliness in younger, working age adults (Barreto et al., 2021). While there is evidence that loneliness is detrimental to employee health (Michaelson et al., 2017), analysis has not been extended to investigate the resulting overarching relationship between loneliness and employment and does not consider the effect of loneliness on individuals that are unemployed. Thus, current understanding of loneliness in working age groups is incomplete. Furthermore, evidence of a relationship between loneliness and factors such as education and employment in adolescence and young adulthood neither extends across working age groups, nor pursues causal explanation (Matthews et al., 2018). More recently, throughout the COVID-19 pandemic, both rates of loneliness and unemployment have attracted increasing government scrutiny. A mapping of the UK illustrates how areas of higher unemployment have higher rates of loneliness during the coronavirus pandemic (Payne, 2021). Overall it is critical to understand the mechanisms and factors related to loneliness and unemployment in order to better understand the health and economic impacts of loneliness in general, as well as to help design interventions and policies to address the immediate impacts of the COVID-19 pandemic. This systematic review addresses these pressing policy questions by critically reviewing studies exploring the relationship between loneliness and employment outcomes.

Loneliness is commonly understood as the subjective or perceived experience of isolation or lack of social support. This perceived deficit can arise from reduced quantity and/or quality of social relationships (Hawkley and Cacioppo, 2010; Peplau, 1982). As loneliness incorporates not only quantity but also quality of social interaction and relationships, a person may experience loneliness without physical social isolation. Research into loneliness and its health related factors is largely concentrated in the older population (Holt-Lunstad et al., 2010; Leigh-Hunt et al., 2017), though the detrimental impact of loneliness can be seen across age groups. Health related consequences of loneliness have been considered in a large number of studies and review articles which observe consistently harmful effects (Heinrich and Gullone, 2006; Holt-Lunstad et al., 2010; Leigh-Hunt et al., 2017; Matthews et al., 2018; Ong et al., 2016; Petitte et al., 2015). These effects include worsened physical health through conditions such as cardiovascular disease, poorer mental health including depression and anxiety, lower wellbeing, negative health behaviours, increased incidence of suicide, and up to 50% increased likelihood of mortality in individuals experiencing loneliness. Overall loneliness has an impact on health comparable to that of smoking and greater than that of obesity and physical inactivity (Holt-Lunstad et al., 2010). There is increasing evidence of loneliness in younger populations with both young and middle-aged people reporting more loneliness than older people (Barreto et al., 2021). Loneliness in working age adults brings additional concerns, potentially impacting educational attainment, employment outcomes and earnings, alongside those effects established in health (Matthews et al., 2018).

It is conceivable that there could be not only an effect of unemployment on loneliness through aspects such as enforced isolation or diminished sense of belonging, but also an effect of loneliness on unemployment through mechanisms such as reduced motivation, performance, or productivity, in addition to poorer health and emotional recognition (Bruehlman-Senecal et al., 2020; Matthews et al., 2018; Michaelson et al., 2017; Ozcelik and Barsade, 2018). The impact of unemployment on health and wellbeing has been investigated in a large number of studies, including review articles, all of which evidence a detrimental effect of unemployment on health (Dooley et al., 1996; Herbig et al., 2013; Jin et al., 1997; Norström et al., 2014; Paul and Moser, 2009; Wanberg, 2012; Wilson and Walker, 1993). These consequences include mental health focused on depression and anxiety, overuse of healthcare resources through increased frequency of physician visits, suicide, and substance abuse including smoking and alcohol. There is also recent evidence that unemployment contributes to poorer quality of life (Norström et al., 2019). These health and socio-economic effects of prolonged unemployment are likely to be persisting (Wadsworth et al., 1999), while loneliness too has both short- and long-term effects on health and well-being (Cacioppo et al., 2003). These inter-related health and socio-economic factors bring complexity to the relationship between loneliness and unemployment. Aspects such as anxiety, depression, optimism, self-esteem, sense of belonging, life satisfaction, coping strategies, health and job-seeking behaviours, and educational attainment and qualifications are just a few of these potentially inter-related or mediating factors which may explain the connection between loneliness and unemployment (Matthews et al., 2018; Payne, 2021).

Given the persistence of both loneliness and unemployment, improved understanding of the mechanisms and factors related to each is key in improving public health across the life course. With increased incidence of both loneliness and unemployment as a result of COVID-19, and pre-existing but often overlooked evidence of a connection between the two experiences, greater understanding of the evidence for the relationship between loneliness and unemployment is required. To date no review has been conducted to untangle this complex, and possibly inter-related or even self-reinforcing (Bruehlman-Senecal et al., 2020) relationship between loneliness and employment. This review seeks to address this gap and develop an understanding of the evidence, strength and direction of the relationship between loneliness and unemployment. Where a relationship is suggested this review will better equip healthcare providers, public policy and employers to support individuals and instigate safeguards to mitigate the impact of loneliness and employment outcomes.

## 2 Methods

This systematic review follows the PRISMA reporting guidelines (Moher et al., 2009). Given the distinct nature of loneliness from social isolation (Heinrich and Gullone, 2006; Peplau, 1982) social isolation was not considered simultaneously in this review.

### 2.1 Identification of studies

Following a brief scoping of existing reviews in loneliness (Boss et al., 2015; Deckx et al., 2018; Dyal and Valente, 2015; Lim et al., 2018; Petitte et al., 2015) and in unemployment (Milner et al., 2013; Norström et al., 2014; Sumner and Gallagher, 2017) we developed a search strategy to identify studies considering any relationship between loneliness and unemployment. The term lonel* was combined with employ* OR unemploy* OR job OR work OR labor/labour to search in title and abstract. We searched the following electronic databases from their inception to December 07, 2020: Embase(Ovid), PsycINFO(Ovid), EconLit(EBSCOhost), MEDLINE(EBSCOhost) and PubMed(NCBI). Titles and abstracts obtained from searches were screened by one reviewer (NM) and potential includes screened by a second reviewer (AML). Full articles were screened for eligibility by one reviewer (NM) and agreed by the second reviewer (AML). Forward and backward citation chasing was conducted on studies identified as included at full-text. These citations were screened following the same process outlined for database searches. Any disagreement was resolved by consensus. Screening was conducted using EndNote X9.

### 2.2 Inclusion criteria

Studies had to evaluate the relationship between loneliness and unemployment. Loneliness was required to be explicitly identified and measured in the analysis. Related concepts such as social isolation or other objective measures were considered insufficient. Studies were to be published in the English language; consider the working age population (aged 16 to 65); and be set in higher income western countries in Europe, North America and Oceania. These were defined by the World Bank as those with a Gross National Income per capita above $12,375 (The World Bank, 2020), and included Europe, Canada, USA, Australia and New Zealand. Included studies were restricted to higher income western countries, in Europe, North America and Oceania, in order to ensure greater consistency in demographics, societal structures, and health and social care systems. Studies were included even if they used data from the same source or sample of respondents. All types of quantitative and qualitative study design were considered.

### 2.3 Exclusion criteria

Studies considering specific populations, such as immigrants or twins, specific occupations, such as dentists, or participants with existing medical conditions, were excluded. Studies were omitted if one or more of the eligibility criteria were not met or if the studies were: methodological, commentaries, letters, editorials or abstracts.

### 2.4 Data extraction

Standardised data forms were used to extract the relevant information from each of the included studies. The data extracted included: author, year, country of data, sample size, age, gender, data source, study design, measurement of loneliness and unemployment, methodology, and key statistical findings. One reviewer (NM) extracted the data and the information extracted was checked by a second reviewer (AML). Results were described in a narrative synthesis and the possibility to perform meta-analysis was explored taking into account the heterogeneity of the studies included with respect to study design and the measures of unemployment and loneliness. Critical appraisal was conducted in line with the Joanna Briggs Institute (JBI) Checklists for Cross-Sectional Studies and for Cohort Studies (Moola et al., 2020). Studies were appraised by one reviewer (NM) and checked by a second (AML). Critical appraisal highlighted any shortcomings or areas of concern within the included papers to ensure balance in the interpretation of findings and highlight areas for improvement in future study on this topic. Consideration was given to any paper with a large number of clear shortcomings before being included in the synthesis.

## 3 Results

### 3.1 Search results

**Fig. 1:**
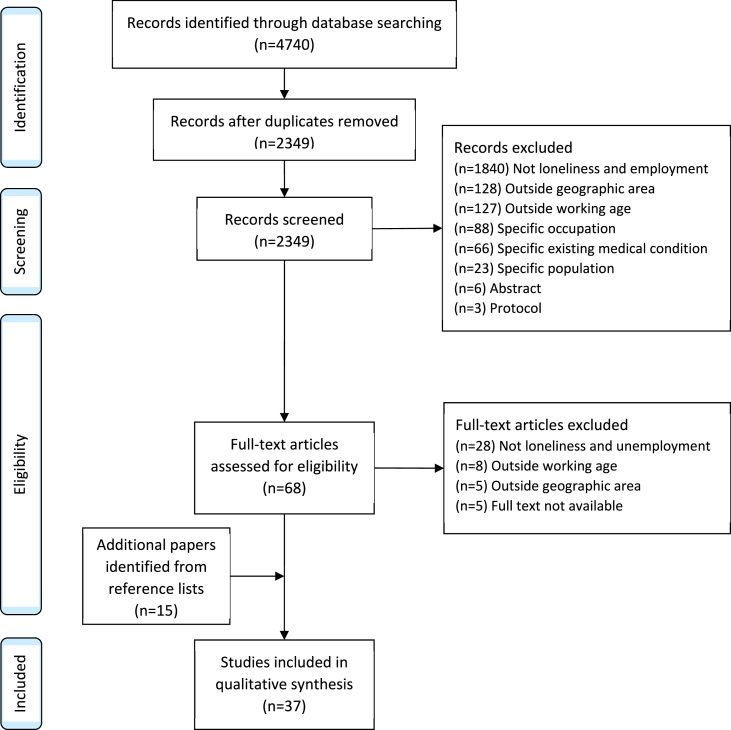
PRISMA diagram.

The electronic searches yielded 4740 hits. When duplicates were removed 2349 unique hits remained. Of these 2281 were excluded through title and abstract screening. Full texts were obtained for 68 studies to check for eligibility. After reviewing full texts 22 studies met the inclusion criteria. Screening of the reference lists added 15 additional papers with a total of 37 studies were included in this review of which 30 were cross-sectional and 7 longitudinal studies. Further details of the study selection process are presented in the PRISMA diagram (Fig. 1).

The opportunity for meta-analysis was explored, however not deemed possible based on the level of heterogeneity between studies in measurement of both unemployment and loneliness (exposures and outcomes) and in study design. While 75% (28/37) studies measured loneliness by a multi-item scale and so could have been combined in meta-analysis, only 30% (11/37) of studies were comparable in terms of employment status measure, being binary employed vs unemployed. The remaining 70% (26/37) of studies had varying definitions and categories defining employment status such that they could not be combined. Furthermore, given the variety in study design, statistical methods, confounders and reporting of results, studies were also not able to be combined by study design. Thus, overall, narrative synthesis was better suited to both manage measurement and methodological heterogeneity, and to capture the complexity of the relationship under consideration.

### 3.2 Critical appraisal

Quality varied across included studies. Of the cross-sectional studies (n = 30) only three fully met the quality criteria with at least one criterion unclear or not fulfilled in the remaining 27 studies. Most cross-sectional studies (29/30) clearly defined their inclusion criteria, though often this was dictated by the chosen data source. The most common area lacking clarity for cross-sectional studies was the identification of confounding factors and the stated strategies to deal with these. Five studies failed to describe study subjects in detail while a further three were unclear. Overall, ten cross-sectional studies had at least one category in which they clearly fell short of the desired quality. Of those, eight studies reported percentages, means or correlation coefficients to indicate a relationship between loneliness and unemployment. Throughout the review, less weight is given to these studies in understanding the relationship such that minor shortfalls in quality did not indicate reason for exclusion. The remaining two cross-sectional studies with a clear ‘no’ in response to the JBI checklist utilised regression analysis and only fell short in description of study subjects thus did not warrant exclusion. Authors described the location and year of the study but demographic details were not provided.

None of the seven cohort studies fully met the quality criteria. Strategies to deal with confounding variables and whether participants were free from exposure at the start of the study were often unclear. Four studies had clear shortcomings in quality, largely surrounding incomplete follow-up without explanation or exploration of this fact. One of these studies (Buecker et al., 2020) also did not employ any strategy to address the incomplete follow up. This poor reporting was not considered grounds for exclusion, however caution is taken throughout this review in presenting a balanced overview of causality and the possibility of bi-directionality providing rationale for further targeted research in this area. Overall, no studies were excluded based on quality appraisal as no shortcomings were considered of critical concern to this review. A summary of study quality for cross-sectional and cohort studies is provided in Table 1, Table 2 respectively.

**Table 1:** Critical appraisal of analytical cross-sectional studies.

Author (Year)	Inclusion criteria defined?	Study subjects described?	Valid, reliable exposure measures?	Objective criteria for condition measurement?	Confounding factors identified?	Stated strategies for confounding?	Valid, reliable outcomes measures?	Appropriate analysis?	Appraisal
Page (1991)	Unclear	Y	Y	Unclear	Unclear	Unclear	Y	Y	Include
Stack (1998)	Y	Y	Y	Unclear	Unclear	Y	Y	Y	Include
Creed (2001)	Y	Unclear	Y	Y	Unclear	N	Y	Y	Include
Lauder (2004)	Y	Y	Y	Y	Unclear	Unclear	Y	Y	Include
Lauder (2006)	Y	Y	Y	Y	Unclear	Unclear	Y	Y	Include
Hawkley (2008)	Y	Y	Y	Y	Unclear	Unclear	Y	Y	Include
Stankunas (2009)	Y	Unclear	Unclear	Y	Unclear	Unclear	Y	Y	Include
Bak (2012)	Y	Y	Unclear	Y	Unclear	Unclear	Y	Y	Include
Fokkema (2013)	Y	Y	Y	Y	Unclear	Unclear	Y	Y	Include
Lykes (2014)	Y	N	Y	Y	Unclear	Unclear	Y	Y	Include
Bosma (2015)	Y	N	Y	Unclear	Y	Y	Y	Unclear	Include
Kearns (2015)	Y	Y	Y	Y	Y	Unclear	Y	Y	Include
Neto (2015)	Y	Y	Y	Unclear	Unclear	Y	Y	Y	Include
Luhmann (2016)	Y	Y	Y	Y	Y	Unclear	Y	Y	Include
Lasgaard (2016)	Y	Y	Y	Y	Unclear	Unclear	Y	Y	Include
Beutel (2017)	Y	Y	Y	Y	N	N	Y	Y	Include
Hawkins-Elder (2018)	Y	Y	Y	Y	Unclear	N	Y	Y	Include
Tong (2019)	Y	N	Unclear	Y	N	N	Y	Y	Include
Bruce (2019)	Y	Y	Y	Y	N	N	Y	Y	Include
Bjelajac (2019)	Y	Y	Y	Y	Unclear	Unclear	Y	Y	Include
Algren (2020)	Y	Y	Y	Y	Y	Y	Y	Y	Include
Emerson (2020)	Y	N	Y	Y	Y	Y	Y	Y	Include
Li (2020)	Y	Y	Y	Y	Y	Y	Y	Y	Include
Lavric (2020)	Y	Y	Y	Y	N	N	Y	Y	Include
Bu (2020b)	Y	Y	Y	Y	Unclear	Unclear	Y	Y	Include
Franssen (2020)	Y	Y	Y	Y	Unclear	Unclear	Y	Y	Include
Cassie (2020)	Y	Unclear	Y	Y	Unclear	Unclear	Y	Y	Include
Wei (2020)	Y	N	Y	Y	Unclear	Unclear	Y	Y	Include
Hawkley (2020)	Y	Y	Y	Y	Unclear	Unclear	Y	Y	Include
Hoffart (2020)	Y	Y	Y	Y	Y	Y	Y	Y	Include

**Table 2:** Critical appraisal of cohort studies.

Author (Year)	Similar groups, same population?	Exposure measures similarly assigned?	Valid, reliable exposure measures?	Confounding factors identified?	Stated strategies for confounding?	Participants free of outcome at start?	Valid, reliable outcome measures?	Follow-up reported and sufficient?	Follow-up complete? If not then explored?	Strategies for incomplete follow-up?	Appropriate analysis?	Appraisal
Kalil (2010)	Y	Y	Y	Y	Y	Unclear	Y	Y	Unclear	Unclear	Y	Include
von Soest (2018)	Y	Y	Y	Y	Unclear	Unclear	Y	Y	Y	Y	Y	Include
Morris (2019)	Y	Y	Y	Y	Y	Y	Y	Y	N	Y	Y	Include
von Soest (2020)	Y	Y	Y	Y	Unclear	Unclear	Y	Y	Y	Y	Y	Include
Buecker (2020)	Y	Y	Y	Y	Y	Y	Y	Y	N	N	Y	Include
Bu (2020a)	Y	Y	Y	Y	Y	N	Y	Y	N	Y	Y	Include
Pagan (2020)	Y	Y	Y	Unclear	Unclear	Unclear	Y	Y	N	Y	Y	Include

### 3.3 Study characteristics

Almost 90% (33/37) of included studies were undertaken in a single country in Europe (n = 22), USA (n = 7), Australia (n = 3) or New Zealand (n = 1). The remaining four studies had a multi-country perspective. Sample size ranged from 114 to 60,341 with seven studies using fewer than 1000 participants. All studies focused on the working age population. One study (von Soest et al., 2020) also included adolescents and many included individuals aged over 65. In these cases mean age remained between 16 and 65. Where reported (20/37), mean age ranged from 21 to 63 years old. Over 65% (25/37) of studies had between 40% and 55% male populations, six studies did not provide descriptive statistics by gender, and six studies were over represented by women with at least 60% of participants identifying as female. The majority (27/37) of studies were carried out using existing national or international databases while 10 studies collected their own data. A large proportion (38%, 14/37) of the studies, included in this review were published in 2020 of which five considered the impact of COVID-19.

### 3.4 Measurement of loneliness

Measurement of loneliness is explicitly defined in all but one study (Bak et al., 2012). Twenty-six studies measured loneliness through an established loneliness scale, one through a scale designed for the survey (Hawkins-Elder et al., 2018), and twelve through a single-item question. Of these, three studies (Bu et al., 2020b; von Soest et al., 2020; von Soest et al., 2018) utilised both a single-item question and a loneliness scale, which is considered the ‘gold standard’ by the UK Office for National Statistics (Osborne et al., 2018). Loneliness scales are considered an indirect measure as they do not explicitly use the term ‘loneliness’ but rather ask about certain feelings that implicitly capture loneliness, such as how often an individual may feel left out, miss the company of others or whether they have people they can trust (Shiovitz-Ezra and Ayalon, 2011). As such, multi-item scales do not require the respondent to openly admit feeling lonely. Meanwhile single-item questions involve a direct question about the respondents' experience or feeling of loneliness and as such require personal recognition of the experience.

In this review 13 studies opted for a form of UCLA Loneliness scale (Russell et al., 1978), 9 studies for a form of De Jong Gierveld scale (de Jong-Gierveld and Kamphuls, 1985), 2 studies used the SELSA (Social and Emotional Loneliness Scale for Adults) (DiTommaso and Spinner, 1993), and 2 the Hughes short scale for measuring loneliness (Hughes et al., 2004). The UCLA loneliness scales varied from 20-item to 3-item versions. The UCLA scale evaluates individual's feelings of loneliness and isolation, and traditionally uses four response categories: never; rarely; sometimes; often (Russell et al., 1978). Studies utilising the De Jong Gierveld scale varied from the more extended 11-item to the 3-item version, and in one case the short scales which evaluate emotional and social loneliness separately were used. The De Jong Gierveld has three response options: no; more or less; yes (de Jong-Gierveld and Kamphuls, 1985). Of those using direct questions to measure loneliness, most (11/12) provided at least 3 response options, most commonly including a selection from; never, rarely, sometimes, often, always. Of these, three studies recoded into a binary format for analysis (Algren et al., 2020; Emerson et al., 2020; Page and Cole, 1991). The remaining study (1/12) provided binary response to feeling of loneliness (Stankunas et al., 2009).

### 3.5 Measurement of employment status

Employment status was either self-reported in a survey (n = 31) or assessed by national register (n = 2). Additionally, one study used both register and survey data (Lasgaard et al., 2016), one included only unemployed individuals (Stankunas et al., 2009), and two studies did not report how data on employment status was collected (Beutel et al., 2017; Tong et al., 2019). Over half of the studies (19/37) included further data collection categories alongside a measurement for being employed or unemployed. These additional categories included being in education; sick/disability; retired; homemaker; economically inactive; benefit claimant; part-time work; military volunteer; rehabilitation; incapacitated.

### 3.6 Study design

Over 80% (30/37) were cross-sectional studies while the remaining 7 used longitudinal data. A third of the studies (13/37) applied weights to account for missing data and/or ensure a representative population. Loneliness was predominantly (80%, 30/37) used as the dependent variable in analysis. Unemployment acted as dependent variable in only 2 studies (Morris, 2019; von Soest et al., 2020). Of the 34 studies utilising regression methodology only 24 used regression methods to evaluate the relationship between loneliness and unemployment. Where conducted, regression standardisation or adjustment changed neither the direction nor statistical significance of the coefficient and resulting relationship identified. The remaining studies (10/34) reported descriptive statistics or correlations for this relationship and used regression for analysis elsewhere. Of the longitudinal studies included, two did not seek to understand directionality (Kalil et al., 2010; Pagan, 2020), three considered the causal impact of employment status on loneliness (Bu et al., 2020a; Buecker et al., 2020; von Soest et al., 2018) and two the reverse of loneliness on employment outcomes (Morris, 2019; von Soest et al., 2020). Further details of study characteristics, data collection and analysis ordered by date of publication can be found in Table 3.

**Table 3:** Study characteristics.

Author (Year)	Study Objectives	Sample Size	Age	Gender	Country	Data Source	Study design	Loneliness		Unemployment		Methodology (loneliness/employment method)
								Measure	Var^a^	Measure	Var^a^	
Page (1991)	To assess the relative strength of demographic variables in explaining frequency of loneliness	8634	3.4% 18–20; 21.9% 21–29; 23.5% 20–29; 15.5% 40–49; 11.3% 50–59; 5.6% 60–64; 6.3% 65–69; 11.5% 70+	44.1% male	USA	Questions administered by the researchers	Cross-sectional	Direct question: how often felt lonely during the past year? Response categories: very often; fairly often; often; sometimes; almost never; never. Recoded as lonely when often, fairly or very; not lonely otherwise	D	Survey data. Response categories: full-time; part-time; retired; unemployed; student; homemaker	I	Descriptive statistics, logistic regression (descriptive statistics, logistic regression)
Stack (1998)	Effect of marital, parental and cohabitation on loneliness, financial satisfaction and reported health	19,652	NR	NR	France, UK, Germany, Italy, Netherlands, Denmark, Belgium, Spain, Ireland, Northern Ireland, USA, Canada, Japan, Australia, Norway, Sweden, Iceland	World Values Survey 1991	Cross-sectional	Direct question: Do you ever feel very lonely? Response categories: 0 = never; 1 = seldom; 2 = sometimes; 3 = frequently.	D	Survey data. Response categories: 1 = unemployed; 0 = all others	I	Ordinary least squares regression (Ordinary least squares regression)
Creed (2001)	To understand the occupational experiences of young people by investigating psychological outcomes	148	Range: 16-26 Mean: 20.54 SD:2.54	50.0% male	Australia	Questions administered by the researchers	Cross-sectional	SELSA Social and Emotional Loneliness Scale for Adults. 14 statements with 5-point Likert-like scale	D	Survey data. Response categories: unemployed with no access to paid work; unemployed with access to some paid work; unemployed with access to regular paid work; full-time employed	I	Descriptive statistics, ANOVA, hierarchical multiple regression (descriptive statistics, hierarchical multiple regression)
Lauder (2004)	To establish the extent of loneliness in a community samples and identify predictors of loneliness	1241	Mean: 45.10 SD:15.44	50.3% male	Australia	Central Queensland Social Survey, 2002	Cross-sectional	11-item De Jong Gierveld Loneliness Scale.	D	Survey data. Response categories: yes/no paid employment previous week	I	Logistic regression (Logistic regression)
Lauder (2006)	To investigate differences in health behaviours in lonely and non-lonely groups	1289	Mean: 46.25 SD:15.61	49.9% male	Australia	Central Queensland Social Survey, 2003	Cross-sectional	11-item De Jong Gierveld Loneliness Scale.	I	Survey data. Response categories: yes/no in paid employment previous week	I	Descriptive statistics, ANCOVA, bivariate analysis, multivariate logistic regression (bivariate analysis)
Hawkley (2008)	To test a conceptual model of loneliness	225	Range: 50-68 Mean: 57.4 SD:4.5	47.6% male	USA	Chicago Health, Aging, and Social Relations Study (CHASRS), wave 1 2002	Cross-sectional	20-item Revised UCLA Loneliness Scale	D	Survey data. Response categories: work full or part time; retired; other	I	Ordinary linear regression (ordinary linear regression)
Stankunas (2009)	Associations between sense of coherence and psychosocial health among unemployed adults	429	Range: 16–64	35.7% male	Lithuania	Questions administered by the researchers in Kaunas Labor Market Office (2005	Cross-sectional	Direct question: Feeling of loneliness? Response categories: 1 = no feeling of loneliness; 2 = feel lonely.	I	All participants were unemployed	I	Student *t*-test, Pearson correlation, ANOVA, logistic regression (descriptive statistic - percentage)
Kalil et al. (2010)	To estimate associations between job insecurity and change in physical and psychological health of older adults	190	Range: 50-68 Mean: 57.41 SD:4.27	47.9% male	USA	Chicago Health, Aging, and Social Relations Study (CHASRS), waves 1–3 2002–2004	Longitudinal	20-item Revised UCLA Loneliness Scale	D	Survey data. Response categories: not working; working	C	Ordinary least squares regression, ordered logit regression, multivariate regression (Ordinary least squares regression)
Bak et al. (2012)	Investigate how stress varies across sociodemographic characteristics in deprived neighbourhoods	1160	Range: 16–104 Mean: 51.63 SD: 17.74	46.2% male	Denmark	Questions administered by the researchers, 2009	Cross-sectional	Not stated	I	Survey data. Response categories: 0 = no unemployment; 1=<3 months; 2 = 3months-1year; 3 = 1–2 years; 4=>2 years. Recoded into 2 categories: 1 = always employed; 2 = unemployed	I	Correlation, hierarchical multiple linear regression (correlation)
Fokkema and Naderi (2013)	To determine whether Turkish older migrants are lonelier than their peers and determine factors accounting for the differences	3742	Range: 18-79 Mean: 63.2 (native); 58.8 (migrant)	48.3% male (native); 50.8% male (migrant)	Germany	German Generations and Gender Survey, waves 2005 and 2006	Cross-sectional	6-item de Jong Gierveld Loneliness Scale	D	Survey data. Response categories: 0 = no paid job; 1 = paid job	I	Descriptive statistics, multivariate regression (multivariate regression)
Lykes and Kemmelmeier (2014)	Examine loneliness as a function of dominant cultural values	38,867	Range: 14–101 Mean: 46.15 SD: 18.21	47.0% male	Austria, Belgium, Bulgaria, Cyprus, Denmark, Estonia, Finland, France, Germany, Hungary, Ireland, Norway, Poland, Portugal, Romania, Russia, Slovakia, Spain, Sweden, Switzerland, The Netherlands, UK	1992 Eurobarometer^c^, and European Social Survey (ESS) Round 3, 2006	Cross-sectional	Direct question: How much of the time during the past week you felt lonely? Response categories: none or almost none of the time, some of the time, most of the time, all or almost all of the time.	D	Survey data. Response categories: 1 = paid work; 0 = no paid work	C	Correlation, generalized linear mixed model, hierarchical linear regression (pairwise correlation, generalized linear mixed model)^b^
Bosma et al. (2015)	Consider whether those with illness and low income have the financial and social resources to cope with disease and life difficulties	24,978	Range: 17–65	NR	The Netherlands	Large epidemiological survey, 2012	Cross-sectional	11-item De Jong-Gierveld scale.	D	Survey data. Response categories: 9 unspecified categories. Coded as employed = highest income, unemployed = lowest income or unemployed	C	Correlation, logistic regression, ordinary least squares regression. (descriptive percentage)
Kearns et al. (2015)	Examine prevalence of loneliness and its association to social contact and support, and its links to self-reported health and wellbeing, in deprived communities	4302	16+	41.1% male	UK	Questions administered by researchers, 2011	Cross-sectional	Direct question: How often been feeling lonely over the last 2 weeks? Response categories: all of the time, often, some of the time, rarely, never. Recoded into 3 categories; frequent; occasional; never	D	Survey data. Response categories: in work; training or education; unemployed; long-term sick; looking after the home; retired	C	Univariable analysis, multivariable multinomial polytomous logistic regression (descriptive statistic - percentage)
Neto (2015)	To examine the relationship between loneliness and psychosocial variables across the adult life span	1209	Range: 18-91 Mean: 38.12 SD:17.49	52.3% male	Portugal	Questions administered by the researchers	Cross-sectional	SELSA-S. Three subscales with 7-point Likert-type scale.	D	Survey data. Response categories: student; worker; unemployed; retired; no answer	I	Descriptive statistics, internal reliability, ANOVA, correlation, hierarchical multiple regression (hierarchical multiple regression)
Luhmann and Hawkley (2016)	Describe and explain age differences in loneliness	16,132	Range: 18–103 Mean: 53.5 SD: 17.2	47.0% male	Germany	German Socio-Economic Panel Study (SOEP) - 2013 wave	Cross-sectional	3-item UCLA Loneliness Scale.	D	Survey data. Response categories: full-time; part-time; voluntary military; unemployed. Recoded into 3 categories: not working at all; working full-time; other	I	Locally weighted scatterplot smoothing curve and confidence bands, omnibus ANOVA tests (descriptive statistic - mean)^b^
Lasgaard et al. (2016)	Identify groups at high risk of loneliness and explore socio-demographic and health-related factors associated to loneliness	33,285	Range: 16–102	49.7% male	Denmark	2013 Danish National Health Survey and Register data	Cross-sectional	3-item UCLA Loneliness scale (Danish version)	D	Survey and register data. Response categories: working; enrolled in education; unemployed; disability pensions; retirement.	I	Multinomial logistic regression, binary logistic regression (Multinomial logistic regression, binary logistic regression)^b^
Beutel et al. (2017)	Determine prevalence and determinants of loneliness in the general population and associations to mental health, health behaviour and healthcare utilisation	15,010	Range: 35–74 Mean: 54.9 SD: 11.1	50.6% male	Germany	Gutenberg Health Study (GHS), baseline data 2007–2012	Cross-sectional	Direct question: I am frequently alone/have few contacts? Responses categories: 0 = no; 1 = yes, but do not suffer; 2 = yes, suffer slightly; 3 = yes, suffer moderately; 4 = yes, strongly suffer. Recoded into 4 categories: never; slight; moderate; severe	D	Not stated	I	Multiple generalized linear models (descriptive statistic – percentage)
Hawkins-Elder et al. (2018)	To identify a typology of loneliness and identify characteristic demographic and psychosocial factors	18,264	Mean: 47.66 SD:14.07	37.2% male	New Zealand	New Zealand Attitudes and Values Study (NZAVS), wave 5 2013	Cross-sectional	NZAVS ‘felt belongingness' measure with three items: ‘I know that people in my life value and accept me’; ‘I feel like an outsider’; ‘I know that people around me share my values and beliefs’	D	Survey data. Response categories: 0 = unemployed; 1 = employed	I	Latent profile analysis (latent profile analysis)
von Soest et al. (2018)	Consider the development and predictors of loneliness in later adulthood	5555	Range: 40–80 Mean: 57.91 SD: 11.11	48.6% male	Norway	NorLAG study, 2002–2007	Longitudinal	Direct question: Do you feel lonely? Response categories: 1 = never, 2 = seldom, 3 = sometimes, 4 = often. 3-item De Jong Gierveld Loneliness Scale.	D	Register data. Response categories: employed; unemployed.	I	Structural equation models, latent loneliness measurement models, latent change score models, linear regression (correlation, linear regression)
Tong et al. (2019)	To determine the associations between community factors and loneliness	1235	18+	NR	USA	Questions administered by the researchers in a primary care waiting room	Cross-sectional	3-item Loneliness Scale (Hughes et al., 2004)	I	Not stated	I	Linear mixed model, Pearson correlation (Pearson correlation)
Morris (2019)	Examine the relationship between loneliness and work disability, and the role of depression as a mediator in the relationship	10,154	Range: 50–65 Mean: 55	NR	Sweden, Denmark, Germany, Belgium, Switzerland, Austria, France, Italy, Spain, Israel, Czech Republic, Slovenia, Estonia, Luxembourg	Survey of Health, Ageing and Retirement in Europe (SHARE) study – 5th (2013) and 6th (2015) waves.	Longitudinal	3-item UCLA Loneliness Scale.	I	Survey data. Baseline response categories: yes; no (work limiting disability). Follow-up response categories: yes; no (being employed at follow up).	D	Pathway model, multivariate logistic regression, binary mediation analysis (logistic regression)^b^
Bruce et al. (2019)	To quantify loneliness and its correlates and inform health behaviour interventions	20,096	18+	38.0% male	USA	Market research survey designed by researchers, 2018	Cross-sectional	3-item UCLA Loneliness Scale	D	Survey data. Response categories: employed full-time, employed part-time, self-employed, unemployed, homemaker, military, retired, student, don't know/not sure.	I	Descriptive statistics, multivariable linear regression, correlation (descriptive statistics)^b^
Bjelajac et al. (2019)	Examine mental health and cognitive functions in older workers accounting for self-assessed health, demographic characteristics and employment status	650	Range: 50–65 Median: 56	NR	Croatia	Survey of Health, Ageing and Retirement in Europe (SHARE) study - wave 6 (2014/15)	Cross-sectional	3-item Short Form Revised-UCLA (R-ULS) Loneliness Scale.	D	Survey data. Response categories: 1 = retired; 2 = employed/self-employed; 3 = unemployed; 4 = permanently sick/disabled; 5 = homemaker or other	I	Logistic regression (descriptive statistic – percentage, logistic regression)
Algren et al. (2020)	Examine whether social isolation and loneliness are associated with health-risk behaviours and modify their relationship to socioeconomic status in deprived neighbourhoods	5113	18+	45.8% male	Denmark	Danish Health and Morbidity Survey 2010 and Deprived Neighbourhood Health Profile Survey 2011	Cross-sectional	First direct question: Are you ever alone, although you would prefer to be together with other people? Response categories: yes; no. Second direct question: felt ‘unwillingly lonely’? Response categories: often; occasionally; rarely; (with additional response of no in Health and Morbidity Study) Recoded into 2 categories: lonely (often); non lonely.	I	Survey data. Response categories: employed; unemployed; disability pensioner; homemaker; long-term sick; in rehabilitation; benefit claimants; non-classifiable. Recoded into 4 categories: employed; unemployed; disability pensioner; other non-employed.	I	Multiple logistic regression (descriptive statistic – percentage)^b^
Emerson et al. (2020)	Evaluate the association between low social connectedness and wellbeing, and test whether disability status moderates the relationship	17,723	Range: 16–64	NR	England	English Community Life Survey (CLS) 2016/17, 2017/18, 2018/19	Cross-sectional	Direct question: How often do you feel lonely? Response categories: often/always; some of the time; occasionally; hardly ever; never. Recoded into 2 categories: lonely (often/always); non lonely.	D	Survey data. Response categories: employed professional; employed intermediate; employed routine; unemployed; economically inactive; full-time student.	I	Poisson regression with robust standard errors, partially adjusted and fully adjusted multivariate models, univariate general linear models (descriptive statistic – percentage)^b^
Li and Wang (2020)	Explore prevalence and predictors of general psychiatric disorders and loneliness	15,530	18+	41.76% male	UK	Understanding Society COVID-19 Study, wave 1 2020	Cross-sectional	Direct question: In the last 4 weeks, how often did you feel lonely? Response categories: hardly ever or never; some of the time; often.	D	Survey data. Response categories: employed; not employed.	C	Univariate analysis, multiple regression – ordinary least squares, binary logistic and ordered logistic regression (descriptive statistics – percentage, ordered logistic regression)^b^
Lavric et al. (2020)	Examine levels of social, emotional and general loneliness in the general population	1189	Range: 18-95 Mean: 46.74 SD: 16.18	49.7% male	Slovenia	Mental Health Literacy Project, 2018–2020	Cross-sectional	De Jong Gierveld short scales	D	Survey data. Response categories: student; employed; unemployed; retired; retired due to disability.	I	T-tests, ANOVA, Hochberg's GT2 and Games-Howell post hoc tests (T-tests, ANOVA)
Bu et al. (2020b)	Compare sociodemographic predictors of loneliness before and during the COVID-19 pandemic	31,064 before; 60,341 during	18+	48.2% male before; 50.2% male during	UK	UK Household Longitudinal Study wave 9 2017–18 (before), and UCL COVID-19 Social Study (during) 21 March - May 20, 2020	Cross-sectional	Direct question: how often felt lonely? Response options: hardly ever/never; sometimes; often 3-item UCLA Loneliness Scale.	D	Survey data. Response categories: employed; unemployed; student; inactive other.	I	Ordinary least squares regression (ordinary least squares regression)^b^
Franssen et al. (2020)	Explore whether factors related to loneliness vary across the adult life span (19–65 years)	26,319	Range: 19–65	45.3% male	The Netherlands	Adult Health Monitor Limburg 2016	Cross-sectional	11-item De Jong Gierveld Loneliness Scale.	D	Survey data. Response categories: retired; unemployed; incapacitated; social assistance; housework; student; employed. Recoded into 2 categories: not having a paid job, having a paid job for at least 1hr per week.	I	Binary logistic regression, multiple logistic regression, multivariate logistic regression (descriptive statistics – percentage, binary logistic regression, multivariate logistic regression)^b^
Cassie et al. (2020)	To examine individual and community level factors associated to social isolation and loneliness	420	Range: 18-90 Mean: 51.77 SD:14.86	24.5% male	USA	Questions administered by the researchers to a convenience sample recruited through social media	Cross-sectional	3-item Loneliness Scale designed for use on telephone survey (Hughes et al., 2004)	D	Survey data. Response categories: 1 = work 30 + hrs per week; 0 = unemployed or work less than 30 + hrs per week	I	Descriptive statistics, linear regression (linear regression)
von Soest et al. (2020)	Understand how substantial sociodemographic, family, social, and personality changes in adolescence and young adulthood may influence loneliness through early and mid-life and how loneliness in adolescence and young adulthood may predict midlife education, work and health outcomes	2602	Range: 13–31	45.9% male	Norway	Young in Norway Study (1992) (T1), 1994 (T2), 1999 (T3), 2005 (T4) and national register data	Longitudinal	Direct question: I feel lonely? Response categories: 1 = never; 2 = rarely; 3 = sometimes; 4 = often. 4-item UCLA Loneliness Scale: (2 emotional and 2 social loneliness items)	I	Register data. Response categories: yes; no (receipt of social or unemployment benefits age 32–35)	D	Latent growth models, probit regression with weighted least square estimator (probit regression with weighted least square estimator)^b^
Wei (2020)	To investigate whether introversion moderates the psychological impact of COVID-19 related circumstantial changes	114	Mean: 30.52 SD:10.02	25.4% male	USA, UK, Canada, Australia, Germany	Questions administered by the researchers	Cross-sectional	De Jong Gierveld Loneliness Scale	D	Survey data. Response categories: 0 = no; 1 = yes recent unemployment due to COVID-19	C	Descriptive statistics, hierarchical regression (descriptive statistics, hierarchical regression)
Buecker et al. (2020)	Investigate the effect of preexisting loneliness on occurrence of major life events, changes in loneliness following major life events, and anticipatory effect of major life events on loneliness	13,945	Range: 16-100 Mean: 44.57 SD: 17.52	45.7% male	The Netherlands	Dutch Longitudinal Internet Studies for the Social Sciences (LISS) panel, 2008–2017 excluding 2012	Longitudinal	6-item De Jong Gierveld Loneliness Scale	D	Survey data. Response categories: yes; no (transition into paid employment, re-employment after unemployment, job loss)	I	1:1 nearest neighbour Propensity Score Matching, generalized additive model (1:1 nearest neighbour Propensity Score Matching, generalized additive model)
Bu et al. (2020a)	Examine how loneliness levels changed in strict lockdown and explore clustering of loneliness trajectories	38,217	18+	49.4% male	UK	UCL COVID-19 Social Study, 2020	Longitudinal	3-item UCLA Loneliness Scale	D	Survey data. Response categories: employed; unemployed; student; inactive other	I	Growth mixture modelling, logistic regression (growth mixture modelling, logistic regression)^b^
Hawkley et al. (2020)	To consider the age distribution of loneliness, explanations for these differences, and evidence for age effects	2477	Range: 18–89 Mean: 50.16 SD:17.06	46.1% male	USA	General Social Survey, 2014, 2018 waves	Cross-sectional	3-item UCLA Loneliness Scale	D	Survey data. Response categories: working full-time or part-time, temporarily without job or unemployed, retired, other e.g. school, housekeeping	I	Locally weighted scatterplot smoothing curve, moderated regression models, multiple regression model (moderated regression models, multiple regression model)^b^
Hoffart et al. (2020)	Evaluate the risk and resilience factors for loneliness in non-pharmacological interventions implemented during COVID-19 and associations between loneliness and psychopathology symptoms	10,061	Range: 18–86 Mean: 36.0 SD: 13.5	21.7% male	Norway	Survey of general adult Norwegian population, 2020	Cross-sectional	UCLA Loneliness Scale-8 (ULS-8)	D	Survey data. Response categories: not stated	I	Hierarchical linear regression, multiple regression (hierarchical linear regression)
Pagan (2020)	To examine the relationships between loneliness, gender, and age for people without and with disabilities	42,569	16+	46.4% male	Germany	German Socio-Economic Panel Study (SOEP) - 2013 and 2017 waves	Longitudinal	3-item UCLA Loneliness Scale	D	Survey data. Response categories: full-time, part-time, non-working	I	Descriptive statistics, locally weighted scatterplot smoothing and kernal-weighted local polynomial regression functions, ordinary least squares model (descriptive statistics, ordinary least squares model)

NR = not reported.

a Var = Variable, D = dependent, I = independent, C = control.

b Weights applied.

c Eurobarometer is older adults so data extracted for ESS study only.

### 3.7 Key findings

The papers can be broadly categorised into exploring the: 1) descriptive statistics on loneliness and unemployment; 2) relationship between loneliness and employment; 3) relationship between loneliness and unemployment; 4) potential for bi-directional relationship; 5) differential effects of impacts by age and gender, and 6) impact of COVID-19 on loneliness and unemployment. Descriptive statistics provide an outline of underlying associations expanded in later sections. Synthesis of the relationship between loneliness and employment is separated from that of unemployment to reflect the differences in experience or onset of loneliness across individuals in and out of work. Where papers consider more than one research category they are included within both categories.

### 3.8 Descriptive statistics on loneliness and unemployment

Most studies (22/37) reported descriptive statistics to indicate experience of loneliness or employment status. These included either the percentage of individuals experiencing loneliness and unemployment or employment (n = 11), mean values (n = 6), or correlation coefficients (n = 5). Of these 22 studies, nine simultaneously reported a linear or logistic regression coefficient. The remaining studies (15/37) presented only regression coefficients (linear, logistic or probit) without this descriptive information.

Across the 11 studies reporting percentages, feelings of loneliness were reported in between 11.1% and 70.2% of unemployed individuals with lower percentages in those who were employed (4.4%–43.3%). Two studies found rates of unemployment rose with severity of loneliness. Unemployment rose from 36.1% in those feeling slight loneliness to 45.5% when severe (Beutel et al., 2017); or from 22.1% with low loneliness to 29.2% with high loneliness (Hawkins-Elder et al., 2018). Four studies (Algren et al., 2020; Emerson et al., 2020; Kearns et al., 2015; Li and Wang, 2020) distinguished individuals who were ‘often’ lonely. Here between 4.4% and 10.2% of employed individuals experienced loneliness ‘often’, compared to between 8.18% and 15.8% of those unemployed. By extending the definition of loneliness to include ‘fairly often’ and ‘sometimes’ the prevalence of loneliness, as measured by percentage, increased up to fourfold. In the 5 studies evaluating the correlation between loneliness and unemployment statistical significance was achieved in 3 studies with correlation coefficients identified between 0.06 (p ≤ 0.001) (von Soest et al., 2018) and 0.49 (p ≤ 0.001) (Tong et al., 2019), indicating an increase in loneliness corresponds to an increase in unemployment. However, this relationship is only weak in almost all studies.

### 3.9 Relationship between loneliness and employment

Twelve studies used regression analysis to consider the relationship between loneliness and being employed or in paid work. Of these, only one study attempted casual analysis through use of a longitudinal dataset (Buecker et al., 2020). In evaluating the relationship between loneliness and being actively employed, linear regression analysis indicated a negative relationship. Four studies yielded negative linear regression coefficients at 5-percent statistical significance. These linear regression coefficients ranged between b = −0.03 (p ≤ 0.05) (Fokkema and Naderi, 2013) and b = −0.218 (p ≤ 0.001) (Lykes and Kemmelmeier, 2014). A further study achieved statistical significance only for a specific subgroup (romantic loneliness) with linear regression coefficient b = −0.14 (p ≤ 0.001) (Neto, 2015). Conversely, only one study identified positive linear regression coefficients (Luhmann and Hawkley, 2016). For both positive and negative linear regression coefficients the magnitude of the relationship between loneliness and paid work was greater in full-time than part-time employment (Luhmann and Hawkley, 2016; Pagan, 2020). Five linear regressions presented negative coefficients but did not achieve statistical significance, which was also the case in causal analysis (Buecker et al., 2020). Logistic regression also suggested reduced experience of loneliness amongst the employed. One study evaluated loneliness and being in employment through multivariate logistic regression and found middle-aged adults in paid work were less likely to be lonely (AOR = 0.71, 95%CI[0.56–0.90]) than their unemployed counterparts. This was reinforced by logistic regression on a sample with mean age 45 which yielded odds ratio OR = 0.73 (95%CI[0.56, 0.94]).

### 3.10 Relationship between loneliness and unemployment

The relationship between loneliness and unemployment was explored through regression analysis in 14 studies. Most studies used cross-sectional data (9/14) while five attempted causal analysis on longitudinal data. Evaluating the relationship between being unemployed and experiencing loneliness, linear regression revealed a statistically significant relationship across the whole sample in only one study (Bu et al., 2020b). Otherwise statistical significance was achieved only in specific subgroups with linear regression coefficients: b = 0.04 (p ≤ 0.01) when considering initial participant status with a loneliness scale as an indirect measure (von Soest et al., 2018); b = 0.31 (p ≤ 0.05) using a direct question for loneliness (von Soest et al., 2020); b = 0.11 (p ≤ 0.001) for only the social loneliness subscale (Neto, 2015); b = 0.338 (p ≤ 0.05) for recent unemployment in a non-USA subsample (Wei, 2020). Meanwhile most studies (4/5) using logistic regression found statistically significant results (Bjelajac et al., 2019; Lasgaard et al., 2016; Li and Wang, 2020; Morris, 2019). One of these studies (Morris, 2019) considered unemployment as the outcome variable in longitudinal data revealing odds ratio OR = 1.252 (p ≤ 0.001) suggesting a 25.2% increase in the likelihood of reporting unemployment, or as the authors describe ‘work disability’, when experiencing loneliness as measured by the UCLA 3-item scale. The remaining studies with statistically significant odds ratios used loneliness as the dependent variable (Bjelajac et al., 2019; Lasgaard et al., 2016; Li and Wang, 2020). They found at least 40% greater odds of reporting loneliness when unemployed based on odds ratios between OR = 1.40 (p ≤ 0.001) and OR = 2.81 (p ≤ 0.05), reaching to OR = 6.93 (p ≤ 0.05) when restricted to experience of severe loneliness. This finding of a greater relationship between loneliness and unemployment in the presence of severe loneliness is reinforced by growth mixture modelling (Bu et al., 2020a).

### 3.11 Potential for bi-directional relationship

Seven studies utilised a longitudinal dataset. Of these, five studies conducted causal analysis in the evaluation of loneliness and unemployment suggesting potential bi-directionality in the relationship. Two studies found loneliness to predict later life unemployment. Morris identified loneliness as predictive of work disability through logistic regression with odds ratio OR = 1.252 (SE = 0.062) (Morris, 2019) while probit regression found loneliness in adolescence and young adulthood predicted higher midlife unemployment with probit regression coefficient b = 0.31, p ≤ 0.05 when using a direct question to measure loneliness (von Soest et al., 2020). Conversely, propensity score matching with a generalized additive model (Buecker et al., 2020) revealed higher reported loneliness following a job loss with linear regression coefficient b = 0.314, p ≤ 0.05, 95%CI[0.111, 0.516]. Unemployment was also observed to positively predict change (von Soest et al., 2018) and growth trajectory (Bu et al., 2020a) of loneliness over time, though without statistical significance. Overall, studies exploring causal inference found higher levels of loneliness following a job loss (Buecker et al., 2020), but also that loneliness was predictive of unemployment in later life (Morris, 2019; von Soest et al., 2020), suggesting a bi-directional relationship.

### 3.12 Differential impacts by age and gender

Three studies (Buecker et al., 2020; Franssen et al., 2020; Lasgaard et al., 2016) evaluated the impact of loneliness and unemployment across different age groups. Lasgaard evaluated experience of severe loneliness and found the greatest relationship to unemployment in those aged 30–44 (OR = 6.64, p ≤ 0,05) with the smallest relationship when aged 16–29 (OR = 3.51, p ≤ 0.05), though still with 351% greater odds of feeling lonely when unemployed. With adjusted odds ratios the greatest effect was rather observed in those age 60–75 (AOR = 4.23, p ≤ 0.05) (Lasgaard et al., 2016). Franssen also found the relationship between loneliness and being in paid work to be age dependent. It was greatest at age 50–65 (OR = 0.56, 95%CI[0.50–0.62]), followed by 19–34 (OR = 0.44, 95%CI[0.37–0.52]) and 35–49 (OR = 0.26, 95%CI[0.22–0.32]). Upon adjusting the odds ratios the greatest effect was observed in those age 19–34 (AOR = 0.72, 95%CI[0.59–0.87]) (Franssen et al., 2020). In full multivariate regression by Franssen, including factors identified as statistically significant at earlier stages, employment was only negatively associated to loneliness in middle-aged adults aged 35–49. Finally, Buecker noted that trajectories after transition into paid employment, reemployment, and after job loss differ depending on age; with a less pronounced increase in loneliness following an employment related event in older than average individuals. Two studies considered differences across gender though found no significant effect on the relationship between loneliness and employment status (von Soest et al., 2018) and little difference in regression coefficients for male and female subsamples (Pagan, 2020).

### 3.13 Impact of COVID-19 on loneliness and unemployment

Five studies, three in the UK (Bu et al., 2020a, b; Li and Wang, 2020), one in Norway (Hoffart et al., 2020), one across multiple countries (Wei, 2020), were conducted during the COVID-19 pandemic. In Norway, the relationship between loneliness and paid work during the COVID-19 pandemic was indicated by linear regression coefficient b = −1.36, but did not achieve statistical significance at the 5% level (Hoffart et al., 2020). One UK based COVID-19 study (Bu et al., 2020b) used linear regression on loneliness and unemployment data from two different surveys. While unemployment remained a persistent risk factor, analysis revealed a drop in the association between loneliness and unemployment from linear regression coefficient b = 0.65 (95%CI[0.5–0.8]) before COVID-19 to b = 0.40 (95%CI[0.25–0.55]) during. Through hierarchical regression, the multi-country study (Wei, 2020) observed a positive and statistically significant relationship between loneliness and recent unemployment due to COVID-19 in non-USA countries (b = 0.338, p ≤ 0.05), but not in the USA. Further details of all key study findings are provided in Table 4.

**Table 4:** Key findings.

Author (year)	% lonely when employed	% lonely when unemployed	% unemployed when lonely	Mean loneliness when employed	Mean loneliness when unemployed	Correlation coefficient (loneliness and unemployment)	Regression coefficient (loneliness and employment)	Regression coefficient (loneliness and unemployment)	Additional Findings
Page and Cole (1991)	Full-time: 9.7%. Part-time: 11.2%	18.8%							Wald statistic employment status = 2.68, p = 0.10. Employment status not statistically significant in logistic regression
Bosma et al. (2015)	6%	20.70%							
Kearns et al. (2015)	Sometimes: 17.7%. Often: 10.2%.	Sometimes: 28.2% Often:15.8%							
Bjelajac et al. (2019)	18%	30%						OR = 1.76* 95%CI[1.13,2.74]	
Algren et al. (2020)	5.1%**	15%**							
Emerson et al. (2020)	Manager: 4.4% [3.6–5.3]. Intermediate: 5.3% [4.2–6.5]. Routine job: 5.8% [4.6–7.3].	11.1% [7.9–15.5]							Marginal mean [95% CI] in respondents without disability. Employment main effect significant at p < 0.001
Li and Wang (2020)	Sometimes: 29.82%*** Often: 6.63%***	Sometimes: 26.73%*** Often: 8.18%***						OR = 1.40*** Standardised OR = 1.16 95%CI[1.20,1.63]	Unstandardised OR = 1.40 (reported), standardised OR = 1.16, SE = 0.11, T-stat = 4.25, p-value = 0.000, 95%CI = [1.20,1.63]
Franssen et al. (2020)	Age 19–34: 34.7%; Age 35–49: 38.4% Age 50–65: 43.3%.	Age 19–34: 54.9%; Age 35–49: 70.2%; Age 50–65: 57.7%.					AOR = 0.71 95%CI [0.56–0.90]		Step 4 multivariate regression analysis model (reported) found being employed only significantly associated to loneliness for early middle-aged adults (age 35–49). Step 3 multiple regression analysis crude (COR) and adjusted (AOR) odds ratios [95% CI]: age 19–34 COR = 0.44[0.37–0.52], AOR = 0.72[0.59–0.87]; age 35–49 COR = 0.26[0.22–0.32], AOR = 0.44[0.36–0.54]; age 50–65 COR = 0.56[0.50–0.62], AOR = 0.66[0.59–0.75].
Stankunas et al. (2009)		35.40%							
Beutel et al. (2017)			Slight: 36.1% Moderate:40.5% Severe: 45.5%						
Hawkins-Elder et al. (2018)			Low loneliness = 22.1%. High loneliness = 29.2%.						Chi-squared to r conversion showed weak effect size r = 0.049 [95%CI 0.034–0.064]. Chi-squared comparison of latent profiles low vs high loneliness χ2 = 19.87, p < 0.001*.
Creed and Reynolds (2001)				28.00 (SD = 8.30)	No access to work: 31.51 (SD = 9.92). Some access to work: 30.42 (SD = 8.97). Regular access to work: 24.93 (SD = 7.40)			No access to work: b = 3.51 (SEE = 1.91; β = 0.18). Some access to work: b = 2.42 (SEE = 2.11; β = 0.11). Regular access to work: b = −3.07 (SEE = 2.14; β = −0.14)	Unemployed with no paid work purported significantly higher levels (p < 0.01) than the unemployed with regular paid work. Occupation made significant contribution in predicting social loneliness: F(3,144) = 3.83, p < 0.05 and accounted for 7% of the variance.
Luhmann and Hawkley (2016)				Full-time: 0.94(SD = 0.68) Part-time: 0.99(SD = 0.72)	1.05(SD = 0.81)		Sample A: full-time b = 0.116***(SE = 0.028), part-time b = 0.075* (SE = 0.032) Sample B: full-time b = 0.067*(SE = 0.029), part-time b = 0.034 (SE = 0.034)		
Bruce et al. (2019)				43.68 (SE = 0.13)	49.03 (SE = 0.27)				
Hoffart et al. (2020)				17.14(SD = 4.73)	19.07(SD = 5.30)		b = −1.36 Standardised β = −0.11(SE = 0.13)		Mean score T-test: t = 15.67, p < 0.001. B = −1.36, SE = 0.13, β = −0.11, t = −10.61*, Part r = −0.10.
Lavric et al. (2020)				General: 2.68**(SD = 1.77). Emotional: 0.80**(SD = 1.01). Social: 1.87(SD = 1.22)	General: 3.51**(SD = 1.87). Emotional: 1.39**(SD = 1.91) Social: 2.13*(SD = 1.12)				Unemployed lonelier than employed for emotional (ΔM = 0.58, SE = 0.11, p < 0.001, 95%CI[0.27,0.89]) and general (ΔM = 0.43, SE = 0.20, p < 0.001, 95%CI[0.44,1.25]) loneliness. No significant difference in social loneliness.
Pagan (2020)				Male: full-time 0.552, part-time 0.040. Female: full-time 0.251; part-time 0.244	Male: 0.407. Female: 0.505		Male: full-time b = −0.152*** (SE = 0.017), part-time b = −0.137*** (SE = 0.026). Female: full-time b = −0.147*** (SE = 0.015); part-time b = −0.135*** (SE = 0.014)		
Bak et al. (2012)						0.112**			For all ages = 0.124**
Lykes and Kemmelmeier (2014)						0.12	b = −0.218*** (SE = 0.025)		Correlation coefficient loneliness and paid work
von Soest et al. (2018)						Baseline: r = 0.06*** (0.08***) Follow up: r = 0.05 (0.07***)		Initial status: β = 0.02(0.04**) Change: β = 0.01(0.02)	Results for direct (indirect) measure of loneliness
Tong et al. (2019)						0.49***			
Wei (2020)						0.156		USA: β = −0.016. Non-USA: β = 0.338*	With inclusion of introversion in the model: USA β = −0.019; Non-USA β = 0.340*
Lauder et al. (2004)							OR = 0.73, p = 0.013 [95%CI 0.56, 0.94], β = −0.321, SE = 0.129		
Kalil et al. (2010)							b = −0.40 (SE = 0.76)		
Fokkema and Naderi (2013)							b = −0.03*		Results for model including health and socioeconomic status. For full model with control variables, risk factors and protective factors b = −0.01
Neto (2015)							Social loneliness: β = 0.03. Family loneliness: β = 0.03. Romantic loneliness: β = −0.14***	Social loneliness: β = 0.11***. Family loneliness: β = 0.06. Romantic loneliness: β = 0.04	Also including subjective well-being variables: employed on social β = 0.05, family β = 0.09**, romantic β = −0.05; unemployed on social β = 0.07*, family β = 0.07*, romantic β = 0.08**.
Cassie et al. (2020)							β = −0.49*		With addition of mental and physical health conditions β = −0.15. With addition of community characteristics β = −0.13
Buecker et al. (2020)							Enter work: b = −0.091 95%CI[-0.230,0.047] Reemployment: b = 0.015 95%CI[-0.221,0.251]	Job loss: b = 0.314* 95%CI[0.111,0.516]	Post-event increase in loneliness after: entering paid work b = −0.002 [-0.149,0.145]; reemployment b = −0.114[-0.355,0.127]; job loss b = 0.451*[0.151,0.751]. Only significant for job loss where immediate short term (1-year) change in loneliness lower in event than control group indicating delay in longer lasting post-event increase.
Hawkley et al. (2020)							Estimate = −0.06, p = 0.461 [95%CI -0.21,0.10]		When also including normative and non-normative predictors the effect of being employed: estimate = −0.01, p = 0.899 [95%CI -0.22, 0.19]. Normative work status as a predictor: estimate = −0.09, p = 0.036 [95%CI -0.18,-0.01] indicating individuals with normative status less lonely than non-normative, though no longer statistically significant when non-normative versions of variables added to the model: estimate = −0.02, p = 0.758 [95%CI -0.16–0.12].
Stack (1998)								β = 0.011	
Hawkley et al. (2008)								Social roles model: b = 1.78 (SE = 1.76). Including all predictors in model: b = 1.58 (SE = 1.64)	
Lasgaard et al. (2016)								Moderate: OR = 2.81*; AOR = 1.81* Severe: OR = 6.93*; AOR = 3.23*	Unadjusted OR (adjusted AOR) severe loneliness different age groups: 16–29yrs 3.51*(2.02*); 30–44yrs 6.64*(3.29*); 45–59yrs 5.92*(2.54*); 60–75yrs 4.17*(4.23*).
Morris (2019)								OR = 1.252*** (SE = 0.062)	Unemployment measured as work disability. Controlling for extra and intra-individual factors (OR = 1.231***, SE = 0.065); full model including health impairment and functional limitation (OR = 1.165**, SE = 0.066); including depression at follow up as mediator (OR = 1.120*, SE = 0.064). Loneliness predictive of work disability at follow up without depression as moderator (β = 0.153***). In full regression model association of loneliness to work disability decreased (β = 0.113*) confirmed by binary-mediation indirect effect of loneliness on work disability (β = 0.028***, 95%CI[0.019, 0.037]) with direct effect (β = 0.054, 95%CI[-0.002,0.109]).
Bu et al. (2020b)								Before COVID-19: b = 0.65 95%CI[0.5–0.8]. During COVID-19: b = 0.4 95%CI[0.25,0.55].	
von Soest et al. (2020)								Direct: β = 0.31*; Emotional: β = 0.09; Social: β = −0.09.	Standardised probit regression coefficient. Slope with control for covariates gender, parental SES and school grades. Without control for covariates: direct measure of loneliness slope = 0.25*; emotional loneliness = 0.05; social loneliness = −0.11.
Bu et al. (2020a)								Low-med lonely: OR = 1.25(SE = 0.30, p = 0.402). Med-high lonely: OR = 1.32(SE = 0.269, p = 0.269). High lonely: OR = 1.75(SE = 0.41, p = 0.064).	Unemployed relative to reference point of being employed without interaction terms: Med-low loneliness OR = 1.26, SE = 0.30, p = 0.388; Med-high OR = 1.33, SE = 0.30, p = 0.261; Highest loneliness OR = 1.75, SE = 0.41, p = 0.066.
Lauder et al. (2006)									Loneliness more common in unemployed: χ2 = 8.083, df = 1, p = 0.004)

*p ≤ 0.05; **p ≤ 0.01; ***p ≤ 0.001.

## 4 Discussion

Interest in the relationship between loneliness and unemployment has increased in recent years with over a third of the included studies published in 2020. There is also a trend towards the use of existing national databases, which in general provides a larger and more varied sample, though at the expense of choice in the measurement of loneliness and employment status. Most studies used self-report employment status and all used a self-report loneliness measure. While loneliness is inherently subjective, and so a self-report measure appropriate for its assessment, there are limitations to self-labelled loneliness, as respondents must personally identify loneliness in themselves. This can be mitigated by using multi-item loneliness scales that capture feelings of loneliness through a wider set of indirect questions. As such, established loneliness scales are preferred over direct questioning, with the De Jong Gierveld and UCLA scales most popular. Only three studies utilised both a direct question and an established multi-item scale to measure loneliness, which is considered the ‘gold standard’ by the UK Office for National Statistics (Osborne et al., 2018). This review identifies a lack of targeted research into the relationship between loneliness and unemployment. Prior to 2018 inferences were limited to correlations by cross-sectional data and underuse of longitudinal datasets. The evaluation of loneliness in relation to unemployment often arises from the inclusion of covariates or controls, and so findings are frequently limited to the descriptive statistics or arise as a by-product in regression analysis. As a result studies are often limited in their evaluation of the nuance and complexity in the relationship between loneliness and unemployment.

As expected, there is evidence that being in paid work may have some protection against experience of loneliness. The greatest benefit is observed in full-time employment given the magnitude of the relationship between loneliness and being employed is consistently greater for those in full-time than part-time work. This suggests the volume of time spent in the workplace can influence loneliness, perhaps due to a sense of community and belonging not always achieved outside of work, and indicates value in promoting workplace engagement for those at greater risk of feeling lonely. Furthermore, one should also consider the impact of workplace environment, including changing working patterns, working from home or frequent changes in jobs, all of which have been increasingly prominent in the COVID-19 pandemic.

Studies evaluating the connection between loneliness and unemployment are consistent in their observation that being unemployed is associated with increased loneliness, particularly with more severe experience of loneliness. Logistic regression, while not determining causality, provided statistically significant findings for the whole sample to reveal at least 40% greater likelihood of unemployed individuals experiencing loneliness (Bjelajac et al., 2019; Bu et al., 2020a; Lasgaard et al., 2016; Li and Wang, 2020) increasing to 693% for those reporting an outcome of severe loneliness (Lasgaard et al., 2016). A greater detrimental effect of more persistent and severe experience of loneliness is also observed in existing research in mental health and executive functioning (Ong et al., 2016; Peplau et al., 1985). This suggests particular importance in providing support for individuals with more severe experience of loneliness and the need to improve understanding of loneliness in relation to employment status could impact both health and economic progression.

This review also identified evidence of at least 25% higher odds of individuals reporting unemployment when lonely (Morris, 2019). However, overall, few studies analysed causal inference and a number while confirming directionality did not achieve statistical significance in evaluating the relationship between loneliness and employment status. This is likely due to the analytical methods and at times sample size available. It could also suggest the need for more focussed analysis controlling for confounders or mediators in the relationship. Employment status is again often included as a sociodemographic control variable rather than a key factor related to loneliness resulting in the relationship being observed as a by-product. This however does reduce concern over publication bias with evidence of a statistically significant relationship arising in the majority of studies. It also promotes the need for future work specifically exploring the complex relationship between loneliness and employment status.

Where statistical significance is achieved in causal inference, studies found higher levels of loneliness following a job loss (Buecker et al., 2020), but also that loneliness was predictive of unemployment (Morris, 2019; von Soest et al., 2020). This suggests potential for a bi-directional relationship between loneliness and unemployment and implies possible self-reinforcing or self-fulfilling relationships where pressures pushing on both factors create a multiplier effect between the two concepts, alongside scope for joint prevention initiatives. This idea of a self-reinforcing loneliness cycle is consistent with existing studies in mental health and behaviour (Bruehlman-Senecal et al., 2020) and is of particular note given the increased rates of both loneliness and unemployment resulting from the COVID-19 pandemic. Due to the limited number of causal inference studies, and the variation in statistical significance which is not achieved by two studies (Bu et al., 2020a; von Soest et al., 2018), these early results should be interpreted with caution with further research needed in this area. While there is potential for direct self-reinforcement between loneliness and unemployment, it is also likely that the cycle could arise from inter-related factors. The nuance and intricacy in the relationship between loneliness and unemployment remains largely unexplored, however some included studies evidenced possible mediation effects surrounding income (Luhmann and Hawkley, 2016) and depression (Morris, 2019). Contributing or mediating factors could include a wide range of inter-related health, social and economic factors linked to both loneliness and unemployment such as anxiety, depression, wellbeing, life satisfaction, executive functioning, and health and job seeking behaviours (Matthews et al., 2018; Ong et al., 2016). Given evidence of a relationship between loneliness and unemployment highlighted in this review, future research should look to further understanding of the complex relationship between loneliness, unemployment and related factors.

This review also indicates that the differential impacts of loneliness on employment have considered age effects, with underreporting of the impacts across gender and ethnicity. The relationship between loneliness and employment status remains prevalent throughout working life. By combining the most at risk identified by Lasgaard and Franssen the greatest association is seen to occur at age 30–34 and age 50–59, consistent with Luhmann's two loneliness peaks, at age 30 and age 60. However, greater effect is observed in the youngest, age 19–34, and the oldest, age 60–75, upon model adjustment. In longitudinal analysis Buecker finds older individuals reported a lower than average increase in loneliness following change in employment status. Findings illustrate the importance of age classification, in particular the oldest (age 65–75) and youngest (age 16–21) where factors such as education and retirement could impact the amount and intensity of paid work. This could also influence the process of defining working age subsamples from the entire population.

As expected, the relationship between loneliness and employment status prevails throughout COVID-19. Evidence from Norway (Hoffart et al., 2020) shows potential for a larger magnitude in the relationship between loneliness and paid work following the onset of the pandemic. However, this study alone, particularly given its lack of statistical significance, is not sufficient for a conclusion to be drawn and as such further research is required. Perhaps unexpected are findings from Bu who did not achieve statistical significance in causal analysis (Bu et al., 2020a) and observed a reduction in the association with the onset of the pandemic (Bu et al., 2020b). There is however no consideration of the impact of the ‘furlough’ scheme, a temporary paid absence scheme introduced by the UK government in response to COVID-19 to prevent mass unemployment, on this finding. This is a key omission given its direct link to unemployment rates. Furthermore, data is restricted to the first three months of UK lockdown (March–May 2020), such that longer-term effects are not considered. On balance the impact of COVID-19, which may persist for a number of years, needs additional consideration. It could be expected that the impact of increased pandemic loneliness on unemployment would become increasingly evident as individuals are encouraged to go back to normalcy and job protection schemes, such as UK furlough, end. With increased incidence of both loneliness and unemployment as a result of the pandemic any association between the two remains of concern.

### 4.1 Limitations

In quantitative studies, which were the most common in this review, reporting guidelines profess the need to explicitly state covariates, such as loneliness and unemployment. However, lack of clarity and consensus in terminology related to both loneliness and unemployment brought limitation to this review. A number of studies may have been inadvertently missed, particularly where unemployment was not stated as a key covariate or sociodemographic control. While comprehensiveness could have been increased by inclusion of additional terms from which to infer employment, such as financial satisfaction, financial resource, work disability, deprivation, life events and sociodemographic, this comes at the expense of precision. We believe our search strategy struck a good balance between pragmatism, comprehensiveness and precision. Furthermore, the electronic search was supplemented by both forward and backward citation chasing. Although, inclusion criteria remained wide, looking for evidence of both loneliness on unemployment and unemployment on loneliness, this provided a more comprehensive overview of the existing literature and greater insight into the complexity of the relationship.

While the potential for meta-analysis was considered in this review, it was concluded that the included studies had insufficient methodological homogeneity to synthesise the results in a meta-analysis. Thus, narrative synthesis was utilised to best capture the nuance of the existing literature on loneliness and unemployment. Included studies contained large variation in study design and reporting of results for standardisation, possibly a result of the lack of targeted research in this area to date. Existing meta-analyses on unemployment and mental health report the need for consistent categories of unemployment, with preference for binary classification to avoid misrepresentation of non-employed persons not seeking work such as students, homemakers or retired individuals (Paul and Moser, 2009). However, studies included in this review contained a wide range of categories for employment status. Given the uncertainty surrounding the impact of using a direct question rather than multi-item loneliness scale in comparing outcomes (Eccles and Qualter, 2021; Masi et al., 2011), it was concluded that while meta-analysis would be possible across different multi-item loneliness scales, studies using a direct question could not be combined with those using a multi-item scale.

### 4.2 Policy implications and future research

This review highlights the need for earlier and more effective recognition of loneliness in both the workplace and outside of work. To achieve these objectives, the relationship between loneliness and unemployment must first be raised amongst employers (Michaelson et al., 2017). As a response, employers should look to ensure good relationship and community, preventing workplace loneliness and associated unemployment. As suggested in recent UK guidance to employers this could be achieved by tackling culture and infrastructure; management; people and networks; work and workplace design; and action in the wider community (Department for Digital et al., 2021). Such measures could also help prevent negative employment outcomes, such as poor employee health, identified in previous literature (Michaelson et al., 2017). Policy objectives should not however be restricted to those in paid work. Increased awareness of unemployment as a risk factor for loneliness or conversely loneliness for unemployment, alongside improved support for newly unemployed and long-term unemployed, could help prevent loneliness and the many detrimental health, social and economic outcomes associated. Providing support through agencies, set up to assist individuals looking or work, could be one such method to reduce or prevent loneliness and/or increase employability to break any self-fulfilling cycles between unemployment and increased loneliness.

The COVID-19 pandemic has increased both unemployment and loneliness (Mental Health Foundation, 2020) highlighting the critical importance of these health limiting issues. COVID-19 has affected both our working lives (economic uncertainty and in some cases the introduction of furlough schemes), and our social lives (lockdowns and social distancing measures). This review raises the finding of a bi-directional relationship between loneliness and unemployment, implying a potential multiplier effect and challenges of possible self-reinforcing or self-fulfilling relationships, alongside the opportunity for joint prevention initiatives. This connection suggests policy makers should not consider loneliness and unemployment in isolation, but focus on both factors simultaneously as they ‘build back better’ following the COVID-19 pandemic. COVID-19 studies included in this review are restricted to early experience of the pandemic where an association of unclear magnitude is observed. Further research into the ongoing experience and aftermath of the pandemic is required with deeper and more focussed attention on the relationship between loneliness and unemployment. In particular, with unemployment set to rise as schemes such as furlough end, effort should be put into understanding and assisting loneliness in those entering unemployment. As the pandemic persists and experience of loneliness and unemployment increases findings suggest a strengthening relationship between the two variables. Furthermore, as loneliness is subjective and not solely due to physical isolation, it will not necessarily be ‘solved’ by mixing after lockdown measures are lifted. Thus, policies should seek to help individuals readjust to a more open society and find ways to reduce any ongoing experience of loneliness.

Further research is also needed to reinforce and confirm the bi-directional relationship between loneliness and unemployment. This is particularly important in the presence of a self-fulfilling multiplier effect, and given the detrimental impact of both loneliness and unemployment on health (Dooley et al., 1996; Heinrich and Gullone, 2006; Herbig et al., 2013; Holt-Lunstad et al., 2010; Leigh-Hunt et al., 2017; Matthews et al., 2018; Norström et al., 2014; Ong et al., 2016; Paul and Moser, 2009). Understanding could be achieved through additional analysis of longitudinal datasets or utilisation of causal methods, such as propensity score matching. This study illustrates how the relationship between loneliness and employment status is often identified as a by-product. Purposeful consideration is required to enable comparison across severity and duration of both loneliness and unemployment, highlighting those at the greatest risk of being caught in a negative cycle. Direct analysis should also consider the inter-related factors or potential mediators in the relationship, such as depression (Morris, 2019) and income (Luhmann and Hawkley, 2016), in order to improve understanding of the complex and nuanced relationship between loneliness and unemployment. Additionally, more research is needed on the longer-term impact of COVID-19 including exploration of government policies, such as the UK furlough scheme, in mitigating the impact of loneliness and employment outcomes. Overall, this review is the first step in understanding the relationship between loneliness and unemployment, presenting evidence of an association and potential bi-directionality. Further research should seek to unpick the complexity of the relationship and improve understanding of bi-directionality.

## 5 Conclusion

Studies show a clear relationship between loneliness and unemployment which extends across the life-course. Individuals who experience loneliness are also more likely to be unemployed. Causality in this relationship is largely under-researched. There are however causal studies suggesting a bi-directional relationship between these outcomes – that job loss leads to loneliness, but also that experiencing loneliness leads to subsequent unemployment. Further targeted research is required to better understand the relationship between loneliness and employment status, particularly around this notion of bi-directionality which can in turn create a self-reinforcing relationship, or multiplier effect, for those most at risk. Future research should also consider inter-related factors in this relationship to better understand its complexity and nuance, particularly regarding the area of mental health. There is a definite trend towards the use of national datasets and inclusion of indirect loneliness measures, or loneliness scales, to assess experience of loneliness. Care should be taken in choice of measure and dichotomisation of loneliness, and also in age group classification as these can influence findings. Research in the area of loneliness and unemployment is of particular importance given the current COVID-19 pandemic. Both loneliness and unemployment are set to rise and, based on the findings of this review, potentially exacerbate and reinforce each other.

## Funding

This work was supported by the 10.13039/501100000272National Institute for Health Research through the Pre-Doctoral Fellowship programme. The funding source had no involvement in study design, writing of the article, or decision to submit for publication. The views expressed in this publication are those of the author(s) and not necessarily those of the National Institute for Health Research or the Department of Health and Social Care.

## Declaration of competing interest

The authors declare they have no conflicts of interest.
